# Laparoscopic gastrectomy reduced peritoneal recurrence in Borrmann type IV gastric cancer: a retrospective cohort study with propensity score matching

**DOI:** 10.1007/s00464-025-11791-5

**Published:** 2025-05-27

**Authors:** Mira Yoo, Yoon Kong, Guan Hong Min, Du-Yeong Hwang, So Hyun Kang, Young Suk Park, Sang-Hoon Ahn, Do Joong Park, Hyung-Ho Kim, Yun-Suhk Suh

**Affiliations:** 1 Department of Surgery, Seoul National University Bundang Hospital, Seongnam, Republic of Korea; 2 Department of Surgery, Seoul National University College of Medicine, Seoul, Republic of Korea; 3 Department of Surgery, Seoul National University Hospital, Seoul, Republic of Korea; 4 Department of Surgery, Chung-Ang University Gwangmyeong Hospital, Gwangmyeong, Republic of Korea; 5 Department of Surgery, Chung-Ang University College of Medicine, Seoul, Republic of Korea

**Keywords:** Laparoscopic gastrectomy, Borrmann type IV, Complication, Survival rate, Recurrence, Retrospective cohort study

## Abstract

**Background:**

Current evidence on the surgical and oncological safety of laparoscopic surgery in patients with Borrmann type IV (B-IV) advanced gastric cancer (AGC) remains insufficient. This study aimed to compare the surgical and prognostic outcomes of laparoscopic gastrectomy (LG) and open gastrectomy (OG) in patients with B-IV AGC.

**Methods:**

Patients with primary B-IV gastric cancer who underwent LG or OG between 2003 and 2019 were retrospectively analyzed. We conducted 1:1 propensity score matching using covariates including sex, age, body mass index, operation type, clinical T and N stages, pathological TNM stage, tumor size, and tumor location. Surgical outcomes, postoperative complications, 5-year survival and recurrence outcomes, and risk factors for peritoneal recurrence were compared between the two groups.

**Results:**

Of 401 patients enrolled, 106 from each of the LG and OG groups were matched, with all standardized differences < 0.1. The LG had significantly fewer wound infections (*P* = 0.029), intra-abdominal abscesses (*P* = 0.035) and a lower peritoneal recurrence rate (5-year cumulative incidence: 48.8% vs. 62.8%, *P* = 0.032; hazard ratio, 0.66; 95% confidence interval, 0.45–0.96) compared to the OG group, along with a trend toward improved 5-year overall survival (LG vs. OG: 37.0% vs. 26.2%, *P* = 0.174; hazard ratio, 0.78; 95% confidence interval, 0.55–1.11). Multivariate analyses revealed a 32.6% decrease in the hazard ratio for peritoneal recurrence in the LG group (*P* = 0.048).

**Conclusions:**

LG significantly reduced peritoneal recurrence with fewer wound and intra-abdominal infectious complications in patients with B-IV AGC.

**Graphical abstract:**

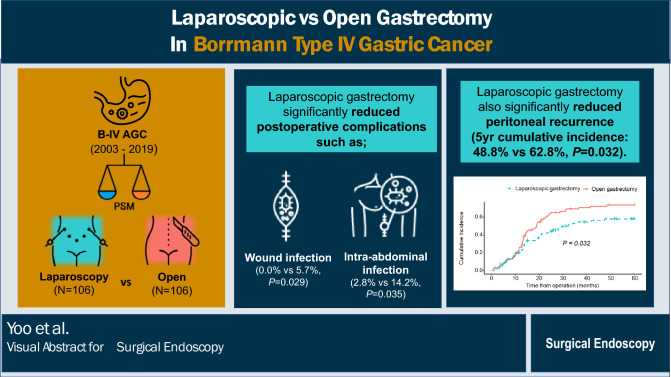

**Supplementary Information:**

The online version contains supplementary material available at 10.1007/s00464-025-11791-5.

The Borrmann classification is a globally recognized system that is extensively used by clinicians to categorize advanced gastric cancer (AGC) [1, 2]. Borrmann type IV (B-IV) gastric cancer is distinguished by its diffuse infiltration throughout the gastric wall, without noticeable ulceration or prominent elevation, accounting for approximately 10–20% of all gastric cancer cases [3, 4]. This subtype is associated with a poor prognosis, with overall 5-year survival rates reported to be less than 30% [4, 5]. Survival outcomes for B-IV gastric cancer patients are heavily influenced by the feasibility of achieving a curative resection [6–8]. Even after curative resection, the 5-year survival rate for B-IV AGC was only 18.1% [7].

Despite the poor prognosis associated with B-IV gastric cancer and the critical role of curative gastrectomy, there have been limited phase III trials or subgroup analyses focusing exclusively on B-IV. This is primarily because B-IV gastric cancer is often excluded from the eligibility criteria owing to factors such as peritoneal seeding at the time of diagnosis [8, 9].

Furthermore, the role of laparoscopic gastrectomy (LG) for B-IV gastric cancer has not been adequately explored, although multiple randomized clinical trials have demonstrated the non-inferiority of LG to open gastrectomy (OG) in terms of surgical and survival outcomes for locally AGC [10, 11]. Previous studies on the safety and efficacy of LG for B-IV gastric cancer have remained controversial. For instance, Kinoshita et al. reported that the hazard ratio (HR) for overall death was relatively higher in the laparoscopic group for B-IV gastric cancer in a subgroup analysis [12]. However, a recent study from the same center suggested that minimally invasive surgery, including robotic surgery, might play an important role in B-IV gastric cancer treatment, provided that appropriate patient selection is based on a multidisciplinary evaluation [13]. Preliminary research at our institution showed a lower peritoneal recurrence rate in the LG group, although this difference was not statistically significant [14].

The objective of this study was to assess the surgical and prognostic outcomes of OG compared to LG in patients with B-IV gastric cancer. Additionally, the study investigated the recurrence patterns and risk factors between these two surgical modalities. This cohort has been reported in line with the Strengthening the Reporting of Cohort Studies in Surgery (STROCSS) guidelines [15].

## Methods

### Study population

We conducted a retrospective cohort analysis of patients diagnosed with primary B-IV AGC who underwent either LG or OG as the initial surgical procedure at Seoul National University Bundang Hospital in the Republic of Korea between June 2003 and December 2019. Patients were excluded based on the following criteria: (1) those who received palliative surgery, (2) individuals with a history of other malignancies, (3) those with previous gastric surgeries, (4) patients who underwent preoperative chemotherapy, (5) surgeries other than distal gastrectomy (DG) or total gastrectomy (TG), and (6) those requiring concomitant resection of non-oncologic organs unrelated to gastric cancer.

Eligible patients were categorized into three groups: open, laparoscopic, and open conversion. The open conversion group, comprising seven patients, was excluded because of the high-risk of bias due to increased complications and poorer prognosis (Supplementary Table S1). To minimize selection bias, we conducted 1:1 propensity score matching (PSM) between the LG and OG groups, using a caliper of 0.1. The covariates for matching were sex, age, preoperative body mass index, operation type (DG or TG), clinical T (cT) stage, clinical N (cN) stage, and pathologic TNM stage (defined according to the eighth edition of the American Joint Committee on Cancer (AJCC) guidelines [16]), tumor size (using the median value of all cases), and tumor location (defined by the highest invasion level of the tumor and divided into four groups: esophagus, upper body, mid body, and lower body). Given the limitations of preoperative staging in Borrmann type IV gastric cancer, pathological TNM stage was included in the propensity score model to better reflect true disease burden and enhance prognostic comparability between groups in survival and recurrence analyses [17].

The study received formal approval from Seoul National University Bundang Hospital Institutional Review Board (IRB) under protocol number B-2304-825-101. Seoul National University Bundang Hospital IRB granted a waiver for informed consent due to the retrospective design and the anonymity of the participants (IRB No. N-2304-825-101). All procedures were carried out in strict accordance with the Declaration of Helsinki and all relevant guidelines and regulations. This study was registered in the Research Registry (unique identifying number: researchregistry11121). The registration was completed prior to submission. Registry link: https://www.researchregistry.com/browse-the-registry#home/registrationdetails/67e0e04c8e5795032d65732b/.

### Intervention and follow-up

All surgeries were performed by board-certified gastrointestinal surgeons with formal training in both open and laparoscopic gastrectomy. Proficiency was defined as completion of at least 50 independent gastrectomies, with the learning curve evaluated based on operative time and complication rates across consecutive cases. Each surgeon performed an average of over 200 gastrectomies annually. All participating surgeons were certified by the Korean Laparoendoscopic Gastrointestinal Surgery Study Group (KLASS) for participation in the KLASS-06 trial (multicenter randomized controlled trial for application of laparoscopic total gastrectomy with lymph node dissection for gastric cancer), a two-arm study comparing laparoscopic versus open radical total gastrectomy with D2 lymph node dissection. This ensured a high level of surgical proficiency and uniform application of operative standards.

At our institution, all surgeons adhere to a standardized operative protocol, including anastomotic techniques for gastrectomies and intraoperative tumor-free practices such as minimal handling of the tumor-bearing stomach. Institutional support included structured training, peer review, and supervised procedures to maintain consistent surgical quality. As such, there was no significant heterogeneity among surgeons in terms of technical approach or adherence to oncologic principles.

The type of gastrectomy, the extent of lymphadenectomy, and the decision to perform splenectomy and intraoperative washing cytology in the LG and OG groups were determined based on the guidelines of the Japanese Gastric Cancer Association (JGCA) and applied consistently to both groups [18–21]. Adjuvant or palliative chemotherapy regimens were administered to eligible patients in both groups by oncology specialists in accordance with the Korean Gastric Cancer Association (KGCA) guidelines [22, 23]. Regardless of whether open or laparoscopic surgery was performed, the standard treatment included 8 cycles of the S-1 for stage II gastric cancer and 8 cycles of XELOX (capecitabine plus oxaliplatin) for stage III gastric cancer as adjuvant chemotherapy. Patients with locally advanced, unresectable, or metastatic gastric cancer were recommended to receive first-line palliative chemotherapy consisting of platinum and fluoropyrimidine [23].

Patients regularly attended outpatient visits after discharge according to the KGCA treatment guidelines. The first visit occurred 2 weeks after surgery, followed by a second visit within 3 months, which included abdominal computed tomography (CT) scans. Subsequent visits continued every 6 months thereafter [23].

### Data acquisition and operational definitions

All demographic data, surgical details, and information on the pathological characteristics of the tumor, lymph nodes (LNs), and recurrence were retrieved from medical records. Survival data were obtained from the medical records of the center and the Korea Statistics Promotion Institute. Complications occurring within 30 days prior to surgery were classified as early, while those occurring after surgery were categorized as late complications. Late complications were observed from 30 days after surgery to the date of an event that was not associated with cancer progression. The severity of each complication was graded according to the Clavien-Dindo classification [24]. Survival was calculated from the date of surgery to the date of any cause of deaths. Recurrence-free survival (RFS) was defined as the period from the date of surgery to either the date of death or first detection of recurrence. Patients with a diagnosis of pathologic M1 were excluded from the survival and recurrence analyses.

### Clinical and pathologic staging

Preoperative clinical staging of the cancer was assessed using abdominal CT and esophagogastroduodenoscopy, along with intraoperative findings such as peritoneal seeding. B-IV gastric cancer was confirmed based on pathological reports. The tumor location was identified according to the findings from the pathological examination.

### Statistical analysis

Categorical variables were analyzed using Pearson’s χ^2^ test or Fisher’s exact test, while continuous variables were compared using Student’s t-test. Survival analysis was conducted using the Kaplan–Meier method along with log-rank test. The prognostic factors for survival and recurrence were analyzed using univariate and multivariate Cox regression models. All *P* values were 2-sided, and *P* value of less than 0.05 was considered statistically significant. Statistical analysis was performed using Statistical Package for the Social Science (SPSS) version 27.0 (IBM Inc., Armonk, NY) and R version 4.3.4 (R foundation for Statistical Computing, Vienna, Austria).

## Results

### Patient characteristics and surgical outcomes

**Fig. 1:**
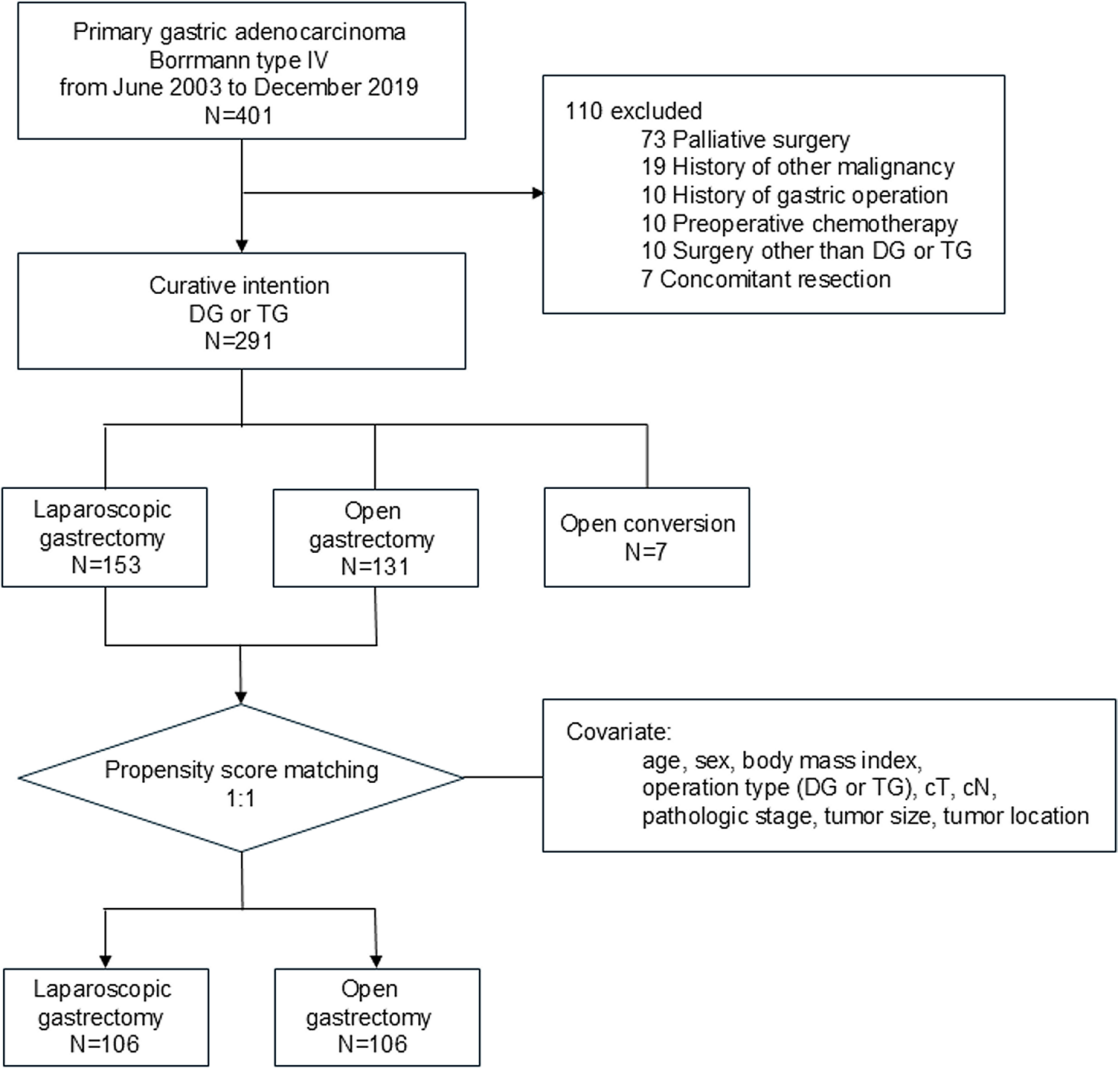
Flowchart of the research strategy

A total of 401 patients were diagnosed with B-IV gastric cancer, of whom the following were subsequently excluded from the study: 73 patients who underwent palliative surgery, 19 with a history of other malignancies, 10 with prior gastric operations, 10 who received neoadjuvant chemotherapy (NAC), 10 who underwent surgery other than distal gastrectomy or total gastrectomy, and 7 who underwent concomitant resection of other organs (Fig. 1). This resulted in 284 patients being eligible for the PSM analysis after excluding 7 cases of open conversion.

The LG and OG groups differed significantly before PSM with respect to cT (standardized mean difference, SMD = 0.468, *P* < 0.001), cN (SMD = −0.258, *P* = 0.026), tumor size (SMD = 0.258, *P* = 0.042), and pathologic TNM stage (SMD = 0.011, *P* = 0.006) (Supplementary Table S2). Among the 284 enrolled patients, 106 patients per group were matched with propensity score adjustment, with all covariates having an SMD < 0.1. The matched population was not significantly different in terms of age, sex, body mass index, American Society of Anesthesiologists score, Charlson Comorbidity Index and prior abdominal operation history in the LG and OG groups.

The clinical characteristics and postoperative outcomes of the study participants before and after PSM are summarized in Table 1 and Supplementary Table S3. After PSM, the LG groups had a shorter hospital stay compared to the OG group (8.7 ± 4.7 days vs. 13.0 ± 12.9, *P* = 0.002) and a longer operation time (255.0 ± 63.2 min vs. 205.4 ± 61.3, *P* < 0.001). The type of surgery performed was similar between the two groups, with total gastrectomy being the most common procedure (75.5% for the LG group, 78.3% for the OG group, *P* = 0.745). The LG group had a larger number of retrieved lymph nodes than the OG group (70.9 ± 24.5 vs 56.8 ± 22.9, *P* < 0.001). Despite this, there was no significant difference in the number of positive lymph nodes between the LG and OG groups (20.6 ± 22.4 vs 17.5 ± 17.7, *P* = 0.262). No significant differences were observed in tumor location, tumor size, radicality, margin involvement, splenectomy, pathological TNM stage, or administration of postoperative chemotherapy.

**Table 1:** Perioperative characteristics in the LG and OG groups (1)

	Before propensity score matching			After propensity score matching		
	LG (*n* = 153)	OG (*n* = 131)	*P*	LG (*n = *106)	OG (*n* = 106)	*P*
Age (year)	59.0 ± 13.2	59.1 ± 13.4	0.982	60.1 ± 1.3	59.1 ± 1.3	0.598
Sex ratio (M:F, %)	57.5: 42.5	52.7: 47.3	0.485	55.7: 44.3	54.7: 45.3	1.000
BMI (kg/m^2^)	23.4 ± 3.4	22.1 ± 3.1	0.001*	22.8 ± 0.3	22.5 ± 0.3	0.543
Hospital stay (day)	8.4 ± 4.6	12.9 ± 12.0	< 0.001*	8.7 ± 4.7	13.0 ± 12.9	0.002*
Operation time (minute)	250.0 ± 64.4	208.2 ± 61.8	< 0.001*	255.0 ± 63.2	205.4 ± 61.3	< 0.001*
Type of operation			0.852			0.745
Distal gastrectomy	36 (23.5%)	33 (25.2%)		26 (24.5%)	23 (21.7%)	
Reconstruction			0.008*			0.017*
Billroth I	2 (5.6%)	4 (12.1%)		1 (3.8%)	4 (17.4%)	
Billroth II	12 (33.3%)	21 (63.6%)		8 (30.8%)	13 (56.5%)	
Roux-en Y	22 (61.1%)	8 (24.2%)		17 (65.4%)	6 (26.1%)	
Total gastrectomy	117 (76.5%)	98 (74.8%)		80 (75.5%)	83 (78.3%)	
Tumor location			0.839			0.860
Esophagus	21 (13.7%)	21 (16.0%)		14 (13.2%)	16 (15.1%)	
Upper body	88 (57.5%)	73 (55.7%)		59 (55.7%)	61 (57.5%)	
Mid body	33 (21.6%)	25 (19.1%)		27 (25.5%)	22 (20.8%)	
Lower body	11 (7.2%)	12 (9.2%)		6 (5.7%)	7 (6.6%)	
Tumor size (cm)	10.4 ± 3.5	11.6 ± 3.7	0.005*	11.1 ± 3.2	11.2 ± 3.5	0.811
Number of retrieved LN	70.0 ± 23.4	57.5 ± 23.0	< 0.001*	70.9 ± 24.5	56.8 ± 22.9	< 0.001*
Number of positive LN	16.7 ± 20.9	18.3 ± 17.7	0.517	20.6 ± 22.4	17.5 ± 17.7	0.262
Splenectomy	20 (13.1%)	34 (26.0%)	0.005*	16 (15.1%)	28 (26.4%)	0.062
pT			0.020*			0.526
T1	2 (1.3%)	0 (0.0%)		0 (0.0%)	0 (0.0%)	
T2	8 (5.2%)	0 (0.0%)		2 (1.9%)	0 (0.0%)	
T3	32 (20.9%)	23 (17.6%)		17 (16.0%)	19 (17.9%)	
T4	111 (72.5%)	108 (82.4%)		87 (82.1%)	87 (82.1%)	
pN			0.004*			0.309
N0	36 (23.5%)	10 (7.6%)		16 (15.1%)	10 (9.4%)	
N1	10 (6.5%)	10 (7.6%)		4 (3.8%)	9 (8.5%)	
N2	21 (13.7%)	25 (19.1%)		16 (15.1%)	19 (17.9%)	
N3	86 (56.2%)	86 (65.6%)		70 (66.0%)	68 (64.2%)	
pM			0.671			0.814
M0	140 (91.5%)	117 (89.3%)		95 (89.6%)	97 (91.5%)	
M1	13 (8.5%)	14 (10.7%)		11 (10.4%)	9 (8.5%)	
pStage			0.006*			0.888
I	5 (3.3%)	0 (0.0%)		1 (0.9%)	0 (0.0%)	
II	35 (22.9%)	14 (10.7%)		14 (13.2%)	14 (13.2%)	
III	100 (65.4%)	103 (78.6%)		80 (75.5%)	83 (78.3%)	
IV	13 (8.5%)	14 (10.7%)		11 (10.4%)	9 (8.5%)	
Postoperative chemotherapy	101 (66.0%)	97 (74.0%)	0.181	83 (78.3%)	75 (70.8%)	0.270

Values are presented as mean ± standardized difference or n (%) unless otherwise indicated

*BMI* indicates body mass index, *LN* lymph node, *pStage* pathologic stage

^*^Statistically significant (*P* < 0.05)

^†^Prior abdominal surgery included appendectomy, cholecystectomy, inguinal hernia repair, cesarean section and ovarian cystectomy

### Postoperative complication

The overall early complication rate after PSM was lower in the LG group (20.8%) than that in the OG group (29.2%), although this difference was not statistically significant (*P* = 0.204; odds ratio (OR), 0.63; 95% confidence interval (CI), 0.34–1.19) (Table 2 and Supplementary Table S4). However, wound complications were significantly lower in the LG group, with no cases reported, compared to 5.7% in the OG group (*P* = 0.029).

**Table 2:** Postoperative complication in the LG and OG groups before and after PSM

	Before Propensity Score Matching			After propensity score matching		
	LG (*n* = 153)	OG (*n* = 131)	*P* value	LG (*n* = 106)	OG (*n* = 106)	*P* value
Early complication	24 (15.7%)	34 (26.0%)	0.046*	22 (20.8%)	31 (29.2%)	0.204
Surgical complication						
Wound	0 (0.0%)	6 (4.6%)	0.024*	0 (0.0%)	6 (5.7%)	0.029*
Bleeding	2 (1.3%)	3 (2.3%)	0.861	2 (1.9%)	1 (0.9%)	1.000
Intra-abdominal Infection	5 (3.3%)	16 (12.2%)	0.008*	3 (2.8%)	15 (14.2%)	0.007*
Anastomosis leakage	2 (1.3%)	3 (2.3%)	0.861	1 (0.9%)	2 (1.9%)	1.000
Stump leakage	0 (0.0%)	1 (0.8%)	0.938	0 (0.0%)	1 (0.9%)	1.000
Postoperative pancreatic fistula	1 (0.7%)	4 (3.1%)	0.280	1 (0.9%)	4 (3.8%)	0.369
Intra-abdominal abscess	2 (1.3%)	8 (6.1%)	0.062	1 (0.9%)	8 (7.5%)	0.035*
Anastomosis stenosis	2 (1.3%)	0 (0.0%)	0.548	2 (1.9%)	0 (0.0%)	0.498
Motility disorder	3 (2.0%)	3 (2.3%)	1.000	3 (2.8%)	2 (1.9%)	1.000
Medical complication						
Pulmonary	7 (4.6%)	2 (1.5%)	0.262	7 (6.6%)	2 (1.9%)	0.170
Urinary	1 (0.7%)	2 (1.5%)	0.892	1 (0.9%)	2 (1.9%)	1.000
Gastrointestinal	1 (0.7%)	0 (0.0%)	1.000	1 (0.9%)	0 (0.0%)	1.000
Hepatobiliary	2 (1.3%)	1 (0.8%)	1.000	2 (1.9%)	1 (0.9%)	1.000
Cardiac	1 (0.7%)	0 (0.0%)	1.000	1 (0.9%)	0 (0.0%)	1.000
Neuropsychiatric	0 (0.0%)	1 (0.8%)	0.938	0 (0.0%)	1 (0.9%)	1.000
Vascular	0 (0.0%)	2 (1.5%)	0.411	0 (0.0%)	1 (0.9%)	1.000
Severity						
Overall complication, C-D ≥ II	23 (15.0%)	28 (21.4%)	0.218	21 (19.8%)	25 (23.6%)	0.617
Surgical complication	11 (7.2%)	21 (16.0%)	0.031*	9 (8.5%)	19 (17.9%)	0.068
Medical complication	12 (7.8%)	7 (5.3%)	0.547	12 (11.3%)	6 (5.7%)	0.218
Overall complication, C-D ≥ IIIa	13 (8.5%)	15 (11.5%)	0.527	12 (11.3%)	16 (15.1%)	0.543
Surgical complication	7 (4.6%)	14 (10.7%)	0.083	6 (5.7%)	15 (14.2%)	0.066
Medical complication	6 (3.9%)	1 (0.8%)	0.184	6 (5.7%)	1 (0.9%)	0.124
Late complication	7 (4.6%)	5 (3.8%)	0.983	5 (4.7%)	4 (3.8%)	1.000
Anastomosis stricture	2 (1.3%)	1 (0.8%)	1.000	1 (0.9%)	1 (0.9%)	1.000
Anastomosis leakage	0 (0.0%)	1 (0.8%)	0.983	0 (0.0%)	1 (0.9%)	1.000
Adhesive ileus	2 (1.3%)	2 (1.5%)	1.000	1 (0.9%)	1 (0.9%)	1.000
Internal hernia	1 (0.7%)	0 (0.0%)	1.000	1 (0.9%)	0 (0.0%)	1.000
Incisional hernia	1 (0.7%)	0 (0.0%)	1.000	1 (0.9%)	0 (0.0%)	1.000
Aorto-esophagojejunostomy fistula	0 (0.0%)	1 (0.8%)	0.938	0 (0.0%)	1 (0.9%)	1.000
Entero-cutaneous fistula	1 (0.7%)	0 (0.0%)	1.000	1 (0.9%)	0 (0.0%)	1.000
Severity						
C-D ≥ II	7 (4.6%)	5 (3.8%)	0.983	5 (4.7%)	4 (3.8%)	1.000
C-D ≥ IIIa	5 (3.3%)	4 (3.1%)	1.000	3 (2.8%)	3 (2.8%)	1.000

Values are presented as n (%)

*LG* indicates laparoscopic gastrectomy, *OG* open gastrectomy, *PSM* propensity score matching, *C-D* Clavien-Dindo classification

^*^Statistically significant (*P* < 0.05)

The rates of other surgical complications, such as bleeding, anastomotic stenosis, and motility disorders, did not differ significantly between the two groups. Notably, intra-abdominal infectious complications, including anastomotic leakage, stump leakage, postoperative pancreatic fistula, and intra-abdominal abscess, were significantly more frequent in the OG group (14.2% vs. 2.8%, *P* = 0.007; OR, 0.18; 95% CI, 0.05–0.63). Among these, intra-abdominal abscesses independently had a lower incidence in the OG group (0.9% vs. 7.5%, *P* = 0.035; OR, 0.12; 95% CI, 0.01–0.95).

Medical complications, including pulmonary, urinary, gastrointestinal, hepatobiliary, cardiac, neuropsychiatric, and vascular, did not differ significantly between the LG and OG groups after PSM.

In terms of severity of overall early complication, based on the Clavien-Dindo classification, there was no significant difference between the LG and OG groups for complications graded as Clavien-Dindo ≥ II (19.8% vs. 23.6%, *P* = 0.617; OR, 0.80; 95% CI, 042–1.54) and Clavien-Dindo ≥ IIIa (11.3% vs. 15.1%, *P* = 0.543; OR, 0.72; 95% CI, 0.32–1.60). Both surgical and medical complications were graded similarly between the two groups, regardless of Clavien-Dindo grade ≥ II or ≥ IIIa.

Late complications were observed in 4.7% of the LG group and 3.8% of the OG group, with no significant difference between the two groups (*P* = 1.000).

### Survival and recurrence outcomes in 5-year after surgery

The median follow-up duration in the LG group was 32.1 months (range, 1.0–60.0), while in the OG group, it was 29.9 months (range, 3.3–60.0).

**Fig. 2:**
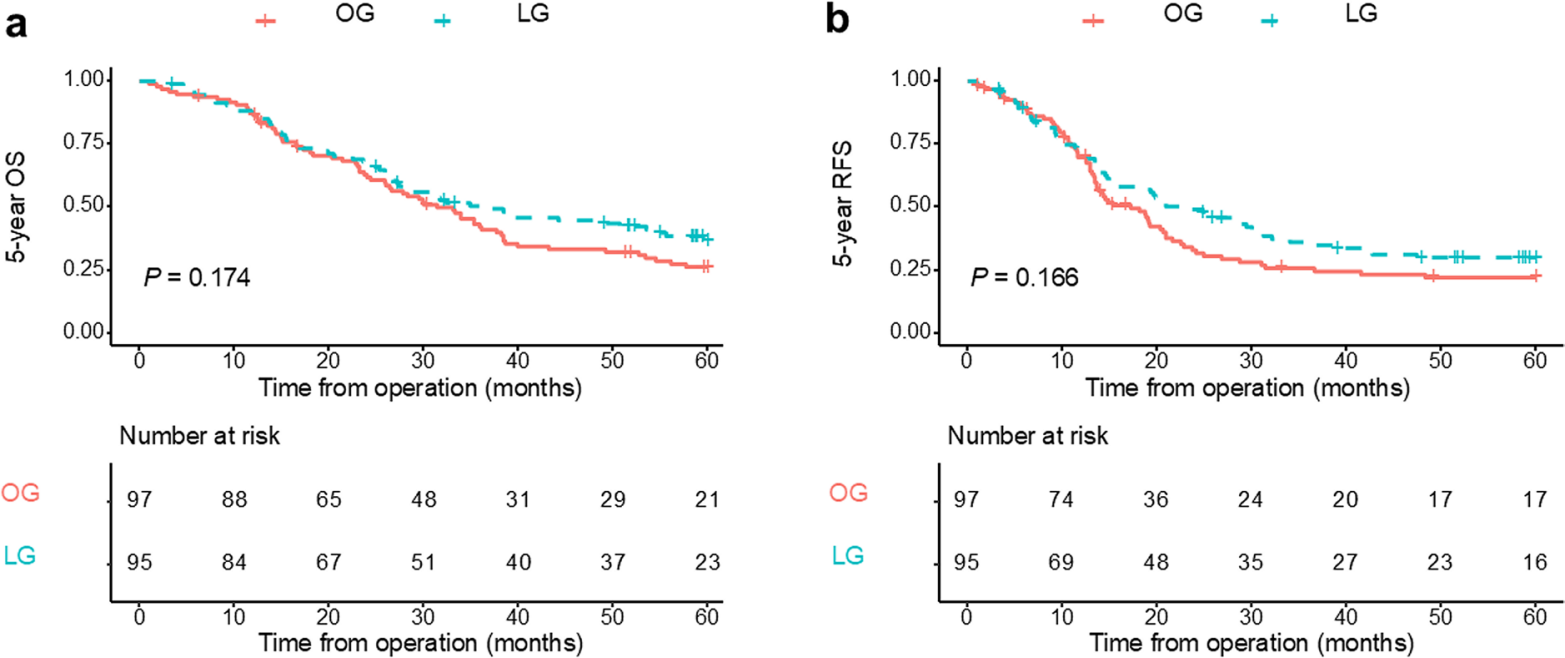
Analysis of 5-year OS (**a**) and 5-year RFS (**b**) in patients treated with LG and OG after PSM

After PSM, the 5-year overall survival (OS) rates were 37.0% for the LG group and 26.2% for the OG group (Fig. 2a). The 5-year recurrence-free survival (RFS) rates were 25.5% and 18.0% in the LG and OG groups, respectively (Fig. 2b). Although both OS and RFS showed a trend favoring the laparoscopic group, these differences did not reach statistical significance (OS: *P* = 0.174; HR, 0.78; 95% CI, 0.55–1.11; RFS: *P* = 0.166; HR, 0.78; 95% CI, 0.56–1.11).

65.3% of patients in the LG group and 71.1% of patients in the OG group experienced recurrence (*P* = 0.472) (Table 3 and Supplementary Table S5). A significant difference was noted in site-specific recurrence, with peritoneal recurrence being substantially higher in the OG group (69.1%) than in the LG group (48.4%; *P* = 0.006; OR, 0.42; 95% CI 0.23–0.76). Other sites of recurrence, such as the anastomosis, retroperitoneum, distant lymph nodes, liver, lung, pleura, bone, and ovary, showed no significant differences between the two groups. Additionally, the incidence of multiple-site recurrences was comparable between the LG (46.3%) and OG (48.5%) groups (*P* = 0.879).

**Table 3:** The first recurrence pattern of the LG and OG groups (after PSM)

	LG (*N* = 95)	OG (*N* = 97)	*P* value
Recurrence	62 (65.3%)	69 (71.1%)	0.472
Anastomosis site	1 (1.1%)	2 (2.1%)	1.000
Peritoneum	46 (48.4%)	67 (69.1%)	0.006*
Retroperitoneum	16 (16.8%)	17 (17.5%)	1.000
Distant lymph node^†^	11 (11.6%)	9 (9.3%)	0.775
Liver	1 (1.1%)	1 (1.0%)	1.000
Lung	2 (2.1%)	2 (2.1%)	1.000
Pleura	2 (2.1%)	2 (2.1%)	1.000
Bone	3 (3.2%)	2 (2.1%)	0.681
Ovary	2 (2.1%)	3 (3.1%)	1.000
Multiple recurrence sites^‡^	44 (46.3%)	47 (48.5%)	0.879

Values are presented as *n* (%)

*LG* indicates laparoscopic gastrectomy, *OG* open gastrectomy, *PSM* propensity score matching

^*^Statistically significant (*P* < 0.05)

^†^Distant lymph nodes are defined as lymph nodes located outside D2 area according to the Japanese Gastric Cancer Association guideline^19^

^‡^Multiple recurrence is defined as the presence of more than one site of recurrence at the time of initial detection

**Fig. 3:**
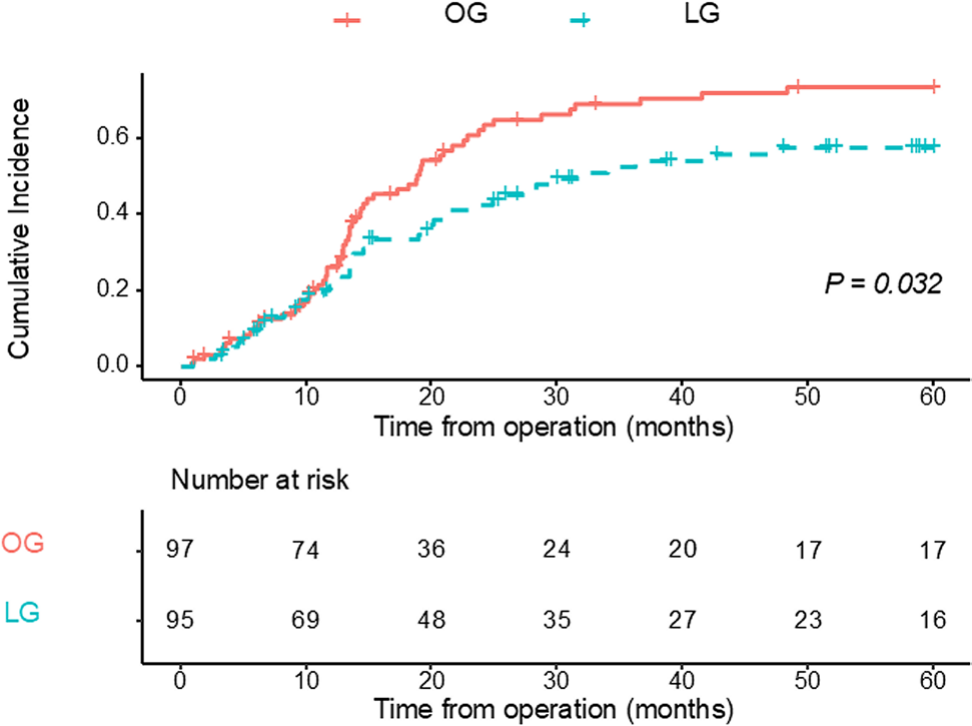
Cumulative incidence of peritoneal seeding after LG and OG

The cumulative incidence of peritoneal recurrence in the LG group was significantly lower (48.8%) than that in the OG group (62.8%; *P* = 0.032; HR 0.66; 95% CI, 0.45–0.96) (Fig. 3).

Univariate Cox regression analysis revealed that patients who underwent LG had a significantly lower risk of peritoneal recurrence than those who underwent OG (HR 0.66, 95% CI 0.45–0.96, *P* = 0.031) (Table 4). The presence of pT4 stage tumors was significantly associated with an increased risk of peritoneal recurrence (HR 2.33, 95% CI 1.33–4.10, *P* = 0.003). Additionally, advanced N stages demonstrated a higher risk of recurrence, with N2 (HR 2.10, 95% CI 1.04–4.24, *P* = 0.040) and N3 (HR 2.23, 95% CI 1.20–4.12, *P* = 0.011). Furthermore, larger tumor size was also independently associated with an elevated risk of recurrence (HR 1.59, 95% CI 1.07–2.34, *P* = 0.020).

**Table 4:** Cox regression analysis of risk factors for peritoneal recurrence (after PSM)

	No. of patients	Univariate analysis		Multivariate analysis	
		HR (95% CI)	*P* value	HR (95% CI)	*P* value
LG (vs OG)	95 (97)	0.66 (0.45–0.96)	0.031*	0.67 (0.46–1.00)	0.048*
Female (vs male)	87 (105)	1.00 (0.68–1.46)	0.997		
Age ≥ 60 years (vs < 60)	97 (95)	0.92 (0.63–1.35)	0.662		
BMI ≥ 25 kg/m^2^ (vs < 25)	86 (106)	1.13 (0.77–1.66)	0.520		
ASA score (vs 1)	(82)				
2	91	0.91 (0.61–1.34)	0.624		
3	18	0.64 (0.29–1.41)	0.271		
CCI (vs ≤ 3)	(89)				
4–5	82	1.25 (0.87–1.77)	0.225		
≥ 6	21	1.24 (0.86–1.78)	0.246		
TG (vs DG)	146 (46)	0.96 (0.77–1.19)	0.694		
pT4 (vs T1 + T2 + T3)	155 (37)	2.33 (1.33–4.10)	0.003*	2.16 (1.22–3.82)	0.008*
pN (vs N0)	(25)				0.027*
N1	13	0.81 (0.26–2.51)	0.713	0.87 (0.28–2.71)	0.805
N2	33	2.10 (1.04–4.24)	0.040*	2.26 (1.11–4.59)	0.025*
N3	121	2.23 (1.20–4.12)	0.011*	1.99 (1.07–3.71)	0.030*
Tumor size ≥ 10.8 cm (vs < 10.8 cm)	104 (88)	1.59 (1.07–2.34)	0.020*	1.44 (0.96–2.15)	0.075
R1 (vs R0)	19 (173)	1.13 (0.62—2.06)	0.692		
Tumor location (vs MB + LB)	(57)				
Esophagus	28	1.50 (0.85–2.63)	0.160		
Upper body	107	0.83 (0.54–1.27)	0.385		
Postoperative chemotherapy No (vs Yes)	52 (140)	1.10 (0.71–1.71)	0.681		

*PSM* indicates propensity score matching, *HR* hazard ratio, *CI* confidence interval, *LG* laparoscopic gastrectomy, *OG* open gastrectomy, *BMI* body mass index, *ASA* American Society of Anesthesiologists, *CCI* Charlson Comorbidity Index, *TG* total gastrectomy, *DG* distal gastrectomy, *MB* mid body, *LB* lower body

^*^Statistically significant (*P* < 0.05)

Multivariate analysis further confirmed the advantage of LG over OG in reducing peritoneal recurrence (HR 0.67, 95% CI 0.46–1.00, *P* = 0.048). The elevated risk associated with pT4 stage tumors remained significant (HR 2.16, 95% CI 1.22–3.82, *P* = 0.008). Higher N stage was a significant predictor of peritoneal recurrence: N2 (HR 2.26, 95% CI 1.11–4.59, *P* = 0.025) and N3 (HR 1.99, 95% CI 1.07–3.71, *P* = 0.030). Although larger tumor size approached significance in the multivariable analysis, it was not a significant predictor (HR 1.44, 95% CI 0.96–2.15, *P* = 0.08).

## Discussion

To the best of our knowledge, this study is the first and most extensive analysis to demonstrate the prognostic benefits of reduced peritoneal recurrence rates with LG compared to OG, for B-IV AGC following comprehensive propensity score matching.

Precise classification and staging of Borrmann type IV gastric cancer remain pivotal yet challenging due to its diffuse infiltration pattern and lack of a discrete tumor mass. While features such as circumferential wall thickening, mucosal rigidity on endoscopy, and poor distensibility on imaging may raise clinical suspicion, the diagnosis of Borrmann type IV and the staging of advanced gastric cancer were ultimately determined by surgical pathology of the gross specimen, which remains the gold standard. Pathologists assessed the resected stomach to determine macroscopic tumor type and depth of invasion. Preoperative clinical assessments and intraoperative findings were used solely as supportive clues—not as definitive diagnostic criteria.

Although Borrmann type IV tumors are typically associated with advanced pathological stages due to their infiltrative behavior, rare cases of early-stage disease can occur. In our cohort, no pT1 cases were identified. Only two patients in the laparoscopy group were classified as pT2, representing 0.9% of the matched population. Among these, one case was staged as pathologic stage I (pT2N0). These rare instances reflect the inherent limitations of preoperative staging and underscore the biological variability and diagnostic challenges of Borrmann type IV gastric cancer. To address these uncertainties, all suspected cases at our institution are evaluated within a multidisciplinary framework, consistent with national and international guidelines [20, 22, 25], where clinical, radiologic, endoscopic, and intraoperative findings are integrated to guide surgical decision-making despite the absence of definitive preoperative pathological confirmation.

B-IV gastric cancer is known for its poor prognosis even after surgical intervention, with high rates of peritoneal recurrence being a major contributing factor [8, 9, 14, 26]. Various efforts to improve survival outcomes, including RCTs investigating NAC, have yielded suboptimal results [5, 27, 28]. One significant issue, particularly following LG or OG for AGC, is the reduced compliance with postoperative chemotherapy, making it difficult for patients to complete the full course of treatment [29, 30]. Additionally, signet ring cell carcinoma, a common histological subtype of B-IV gastric cancer, has demonstrated poor responsiveness to chemotherapy at the molecular level [31–33]. Although NAC and conversion therapy are increasingly utilized in the treatment of advanced gastric cancer, their role in Borrmann type IV disease remains controversial due to the tumor’s unique biological characteristics, including diffuse infiltration, poor differentiation, and a high propensity for peritoneal dissemination. In East Asia, particularly Korea and Japan, NAC is not widely adopted as a standard approach for resectable gastric cancer. This is largely influenced by the results of previous studies such as the JCOG0501 trial, which investigated the use of NAC in patients with type IV and large type III tumors but did not demonstrate a significant survival benefit over upfront surgery [34]. While more recent trials, including PRODIGY and RESOLVE, have reported favorable outcomes for perioperative chemotherapy in specific patient groups, NAC remains cautiously applied and has not been incorporated into routine practice for Borrmann type IV tumors in the region [35, 36].

To reduce treatment-related heterogeneity and allow a focused comparison of surgical modality alone, patients who received NAC or conversion therapy were excluded from our analysis. This approach enabled a more direct evaluation of the oncologic and perioperative outcomes associated with laparoscopic versus open gastrectomy. It is also noteworthy that only 10 out of 401 patients (2.5%) initially assessed had received NAC, reflecting historical treatment patterns at our institution during the study period (2003–2019). While this enhances the internal validity of our findings, we acknowledge that it may limit generalizability in the context of evolving global treatment strategies. Future prospective studies are warranted to evaluate the role of minimally invasive surgery in patients undergoing NAC or conversion therapy for Borrmann type IV gastric cancer.

Given these difficulties, there is a pressing need to establish surgical treatment strategies for B-IV. This matched comparative study suggests that LG may offer oncologic and perioperative advantages over OG in this aggressive subtype. In addition to a shorter hospital stay and a higher number of retrieved lymph nodes, the LG group demonstrated fewer wound infections and intra-abdominal infectious complications. Notably, LG was also associated with a significantly lower cumulative incidence of peritoneal recurrence, which underscores its potential benefit even in advanced and infiltrative gastric cancers such as B-IV.

Contrary to longstanding concerns that minimally invasive surgery may increase the risk of peritoneal spread in advanced cancers, our findings suggest that LG may in fact offer protective advantages [12, 37, 38]. This could be attributed to several technical and biological factors: improved visualization and precision during dissection, gentle tumor manipulation, and the creation of a pneumoperitoneum with carbon dioxide, which has been hypothesized to minimize tumor cell aerosolization and implantation by maintaining a relatively closed and stable operative environment [39, 40]. These factors, combined with a lower systemic inflammatory response and enhanced perioperative control, may help reduce the risk of peritoneal dissemination during surgery [41–45]. Nonetheless, further investigation is warranted to clarify these mechanisms and to determine whether the observed association between LG and reduced peritoneal recurrence reflects a true causal relationship.

Our data demonstrated that since 2010, the number of LG cases for B-IV gastric cancer has surpassed that of OG cases (Supplementary Fig. S1). This shift reflects both the maturation and increased adoption of laparoscopic techniques, despite the initial shortage of experienced surgeons. This trend highlights the evolving expertise and improved outcomes associated with LG [46]. In contrast, OG had already reached a proficiency level where even challenging cases, such as B-IV gastric cancer, could be effectively managed, making it a suitable control group for this study [47].

Given these dynamic changes in surgical practice, we further conducted a subgroup analysis stratified by surgical era to assess whether the oncologic outcomes associated with LG have evolved over time. Stratification by year of surgery—2003–2007, 2008–2012, and 2013–2019—allowed us to understand transitional period according to the clinical adoption of pivotal adjuvant chemotherapy trials (ACTS-GC and CLASSIC), as well as the incremental refinement of minimally invasive surgical techniques [48, 49].

Within each era, LG and OG were compared with respect to 5-year OS, RFS, and peritoneal recurrence (Supplementary Table S6, Figs. S2, S3, and S4). LG consistently demonstrated favorable trend of outcomes across all eras, with a significantly reduced peritoneal recurrence in the most recent period (2013–2019) (45.3% vs. 67.5%, *P* = 0.032). Furthermore, the proportion of patients receiving adjuvant chemotherapy remained statistically comparable between LG and OG groups throughout the study period (all *P* > 0.05), supporting the interpretation that the observed benefits of LG were not confounded by disparities in systemic therapy.

It is important to contextualize these findings within the broader timeline of clinical practice. The implementation of evidence-based adjuvant chemotherapy following ACTS-GC and CLASSIC was not immediate and was influenced by institutional policies, training, and reimbursement frameworks. Thus, a temporal mismatch exists between the evolution of surgical technique and the uptake of postoperative chemotherapy, particularly in the earlier periods. This further justifies the use of era-based subgrouping to reduce bias stemming from historical variability in practice patterns.

Taken together, our findings suggest that the benefits of LG, particularly in reducing peritoneal recurrence, have become more evident in recent years as surgical expertise and perioperative oncologic strategies have matured. While definitive causal conclusions cannot be drawn, the results underscore the oncologic safety and potential advantage of LG in appropriately selected patients with Borrmann type IV gastric cancer, and they support its consideration in the design of future prospective trials.

Our findings are also supported by recent evidence demonstrating the oncologic safety of laparoscopic gastrectomy in advanced and biologically aggressive gastric cancer. In a large multicenter randomized controlled trial, laparoscopy-assisted distal gastrectomy was shown to be noninferior to open surgery in terms of 5-year relapse-free and overall survival, with no significant difference in postoperative morbidity when performed by qualified surgeons [50]. Furthermore, a multicenter retrospective study of scirrhous gastric cancer reported comparable long-term outcomes between laparoscopic and open approaches, underscoring the feasibility and safety of minimally invasive techniques even in this high-risk subgroup [51].

Despite the valuable insights provided by our study, several limitations should be acknowledged. First, the retrospective design inherently carries the risk of selection bias and unmeasured confounders, even with the application of propensity score matching. Factors such as tumor molecular characteristics, surgeon-specific technical variations, and institutional protocols may have influenced outcomes but were not captured in our dataset. Second, temporal bias was unavoidable, as laparoscopic gastrectomy cases were predominantly performed in more recent years when advancements in surgical technology, perioperative care, and adjuvant therapy may have contributed to improved outcomes, independent of the surgical approach itself. While this reflects natural evolving clinical practice rather than a methodological flaw, it does limit causal inference. Third, although the observational period extended up to 60 months, the median follow-up duration (LG: 32.1 months vs. OG: 29.9 months) may limit the assessment of long-term outcomes. However, this is largely reflective of the natural course of Borrmann type IV gastric cancer, which is characterized by aggressive progression, early recurrence, and high mortality—leading to a substantial number of events within the first few years after surgery. Fourth, while the matched sample size was modest (n = 106 per group), this study represents one of the largest single-center cohorts focused exclusively on Borrmann type IV gastric cancer. Despite limited statistical power to detect subtle survival differences, the study offers meaningful real-world insights into this rare and aggressive disease subtype. Future prospective, multicenter studies with standardized protocols and comprehensive data collection are warranted to validate these findings and further clarify the role of laparoscopic surgery in Borrmann type IV gastric cancer. Such efforts will be critical to establishing its efficacy and ensuring its safe integration into clinical practice.

## Conclusion

Laparoscopic gastrectomy appeared to be superior to open gastrectomy in reducing the risk of peritoneal recurrence, with fewer wound and intra-abdominal infectious complications in patients with B-IV gastric cancer.

## Supplementary Information

Below is the link to the electronic supplementary material.

Supplementary file1 (DOCX 1255 KB)

## Data Availability

The data that support the findings of this study are not publicly available due to institutional restrictions and patient confidentiality.
