# Supplementary material for: Laparoscopic gastrectomy reduced peritoneal recurrence in Borrmann type IV gastric cancer: a retrospective cohort study with propensity score matching
Source: Surg Endosc. 2025 May 27;39(7):4316–28. doi: 10.1007/s00464-025-11791-5 (PMC12222315; doi:10.1007/s00464-025-11791-5)
Supplement: Supplementary file 1 — Supplementary file1 (DOCX 1255 KB) [file 464_2025_11791_MOESM1_ESM.docx]

| **Supplementary Table 1** Characteristics of the patients who underwent open-conversion gastrectomy | | | | | | | | | | | | |
| --- | --- | --- | --- | --- | --- | --- | --- | --- | --- | --- | --- | --- |
| Patient No. | Age | Sex | BMI | Type of Converted Operation | Reason of Open Conversion | pT | pN | pM | Hospital Stay | Complication | 5yr Recurrence | 5yr Mortality |
| 1 | 68 | Male | 25.7 | TG | Extensive tumor | T3 | N1 | M0 | 9 | None | None | None |
| 2 | 40 | Male | 22.6 | TG | Extensive tumor | T4a | N3b | M0 | 6 | None | 22m | 29m |
| 3 | 69 | Male | 26.4 | TG | Bleeding | T3 | N1 | M0 | 8 | None | None | None |
| 4 | 46 | Male | 24.1 | TG | Bleeding | T4b(TC) | N3a | M1 | 27 | Intra-abdominal abscess | - | 7m |
| 5 | 43 | Female | 27.6 | TG | Extensive tumor | T4b(TC) | N3a | M0 | 11 | Urinary infection | None | None |
| 6 | 69 | Male | 20.4 | TG | Extensive tumor | T4b(TC) | N3b | M0 | 9 | None | 6m | 13m |
| 7 | 64 | Female | 19.8 | TG | Extensive tumor | T4b(TC) | N3b | M1 | 8 | None | - | 6m |
| The unit of age is year; kg/m^2^ for BMI, day for hospital stay, m(month) for postoperative period at recurrence or mortality. | | | | | | | | | | | | |
| BMI indicates body mass index; TG, total gastrectomy; TC, transverse colon (invaded organ). | | | | | | | | | | | | |

| **Supplementary Table 2** Before and after propensity score matching | | | | | | | | | |
| --- | --- | --- | --- | --- | --- | --- | --- | --- | --- |
| Covariates | Before propensity score matching | | | |  | After propensity score matching | | | |
|  | LG (n=153) | OG (n=131) | *P* | SMD |  | LG (n=106) | OG (n=106) | *P* | SMD |
| Sex: Female | 65 (42.5%) | 62 (47.3%) | 0.485 | -0.098* |  | 47 (44.3%) | 48 (45.3%) | 1.000 | -0.019* |
| Age: ≥60 years | 73 (47.7%) | 67 (51.1%) | 0.647 | -0.069* |  | 52 (49.1%) | 55 (51.9%) | 0.784 | -0.057* |
| BMI:≥25.0 kg/m^2^ | 79 (51.6%) | 52 (39.7%) | 0.058 | 0.239 |  | 46 (43.4%) | 48 (45.3%) | 0.890 | -0.038* |
| cT |  |  |  | -0.468 |  |  |  | 0.394 | -0.055* |
| cT0+T1 | 18 (11.8%) | 1 (0.8%) | < 0.001* |  |  | 4 (3.8%) | 1 (0.9%) |  |  |
| cT2+cT3 | 61 (39.9%) | 39 (29.8%) |  |  |  | 35 (33.0%) | 37 (34.9%) |  |  |
| cT4a+T4b | 74 (48.4%) | 91 (69.5%) |  |  |  | 67 (63.2%) | 68 (64.2%) |  |  |
| cN: cN+ | 97 (63.4%) | 100 (76.3%) | 0.026* | -0.258 |  | 75 (70.8%) | 80 (75.%) | 0.536 | 0.000* |
| Tumor location |  |  | 0.839 | -0.351 |  |  |  | 0.860 | 0.061* |
| Esophagus | 21 (13.7%) | 21 (16.0%) |  |  |  | 14 (13.2%) | 16 (15.1%) |  |  |
| Upper body | 88 (57.5%) | 73 (55.7%) |  |  |  | 59 (55.7%) | 61 (57.5%) |  |  |
| Mid body | 33 (21.6%) | 25 (19.1%) |  |  |  | 27 (25.5%) | 22 (20.8%) |  |  |
| Lower body | 11 (7.2%) | 12 (9.2%) |  |  |  | 6 (5.7%) | 7 (6.6%) |  |  |
| Type of operation |  |  | 0.844 | 0.040* |  |  |  | 0.745 | 0.021* |
| DG | 36 (23.5%) | 33 (25.2%) |  |  |  | 26 (24.5%) | 23 (21.7%) |  |  |
| TG | 117 (76.5%) | 98 (74.8%) |  |  |  | 80 (75.5%) | 83 (78.3%) |  |  |
| Tumor size: ≥10.8cm | 68 (44.4%) | 75 (57.3%) | 0.042* | -0.258 |  | 58 (54.7%) | 58 (54.7%) | 1.000 | 0.000* |
| Pathologic TNM stage |  |  | 0.006* | 0.011* |  |  |  | 0.740 | 0.000* |
| I | 5 (3.3%) | 0 (0.0%) |  |  |  | 1 (0.9%) | 0 (0.0%) |  |  |
| II | 35 (22.9%) | 14 (10.7%) |  |  |  | 14 (13.2%) | 14 (13.2%) |  |  |
| III | 100 (65.4%) | 103 (78.6%) |  |  |  | 80 (75.5%) | 83 (78.3%) |  |  |
| IV | 13 (8.5%) | 14 (10.7%) |  |  |  | 11 (10.4%) | 9 (8.5%) |  |  |
| Values are presented as n (%) unless otherwise indicated. | | | | | | | | | |
| *Statistically significant (*P*<0.05 or \|SMD\|<0.1). | | | | | | | | | |
| LG indicates laparoscopic gastrectomy; OG, open gastrectomy; SMD, standardized mean difference; BMI, body mass index; DG, distal gastrectomy; TG, total gastrectomy; TNM, tumor-node-metastasis. | | | | | | | | | |

| **Supplementary Table 3** Perioperative characteristics in the LG and OG groups (2) | | | | | | | |
| --- | --- | --- | --- | --- | --- | --- | --- |
|  | Before propensity score matching | | |  | After propensity score matching | | |
|  | LG (n=153) | OG (n=131) | *P* |  | LG (n=106) | OG (n=106) | *P* |
| Age (year) | 59.0 ± 13.2 | 59.1 ± 13.4 | 0.982 |  | 60.1 ± 1.3 | 59.1 ± 1.3 | 0.598 |
| Sex ratio  (M:F, %) | 57.5 : 42.5 | 52.7 : 47.3 | 0.485 |  | 55.7 : 44.3 | 54.7 : 45.3 | 1.000 |
| BMI (kg/m^2^) | 23.4 ± 3.4 | 22.1 ± 3.1 | 0.001* |  | 22.8 ± 0.3 | 22.5 ± 0.3 | 0.543 |
| ASA score |  |  | 0.457 |  |  |  | 0.276 |
| I | 59 (38.6%) | 60 (45.8%) |  |  | 40 (37.7%) | 47 (44.3%) |  |
| II | 81 (52.9%) | 62 (47.3%) |  |  | 54 (50.9%) | 53 (50.0%) |  |
| III | 13 (8.5%) | 9 (6.9%) |  |  | 12 (11.3%) | 6 (5.7%) |  |
| CCI |  |  | 0.937 |  |  |  | 0.643 |
| 1 ~ 2 | 36 (23.5%) | 33 (25.2%) |  |  | 22 (20.8%) | 27 (25.5%) |  |
| 3 ~ 4 | 68 (44.4%) | 58 (44.3%) |  |  | 48 (45.3%) | 48 (45.3%) |  |
| ≥5 | 49 (32.0%) | 40 (30.5%) |  |  | 36 (34.0%) | 31 (29.2%) |  |
| Prior abdominal  surgery^†^ | 31 (20.3%) | 16 (12.2%) | 0.097 |  | 23 (21.7%) | 15 (14.2%) | 0.210 |
| Hospital  stay (day) | 8.4 ± 4.6 | 12.9 ± 12.0 | < 0.001* |  | 8.7 ± 4.7 | 13.0 ± 12.9 | 0.002* |
| Operation time  (minute) | 250.0 ± 64.4 | 208.2 ± 61.8 | < 0.001* |  | 255.0 ± 63.2 | 205.4 ± 61.3 | < 0.001* |
| Type of  operation |  |  | 0.852 |  |  |  | 0.745 |
| Distal gastrectomy | 36 (23.5%) | 33 (25.2%) |  |  | 26 (24.5%) | 23 (21.7%) |  |
| Reconstruction |  |  | 0.008* |  |  |  | 0.017* |
| Billroth I | 2 (5.6%) | 4 (12.1%) |  |  | 1 (3.8%) | 4 (17.4%) |  |
| Billroth II | 12 (33.3%) | 21 (63.6%) |  |  | 8 (30.8%) | 13 (56.5%) |  |
| Roux-en Y | 22 (61.1%) | 8 (24.2%) |  |  | 17 (65.4%) | 6 (26.1%) |  |
| Total gastrectomy | 117 (76.5%) | 98 (74.8%) |  |  | 80 (75.5%) | 83 (78.3%) |  |
| Tumor location |  |  | 0.839 |  |  |  | 0.860 |
| Esophagus | 21 (13.7%) | 21 (16.0%) |  |  | 14 (13.2%) | 16 (15.1%) |  |
| Upper body | 88 (57.5%) | 73 (55.7%) |  |  | 59 (55.7%) | 61 (57.5%) |  |
| Mid body | 33 (21.6%) | 25 (19.1%) |  |  | 27 (25.5%) | 22 (20.8%) |  |
| Lower body | 11 (7.2%) | 12 (9.2%) |  |  | 6 (5.7%) | 7 (6.6%) |  |
| Tumor size (cm) | 10.4 ± 3.5 | 11.6 ± 3.7 | 0.005* |  | 11.1 ± 3.2 | 11.2 ± 3.5 | 0.811 |
| Number of  retrieved LN | 70.0 ± 23.4 | 57.5 ± 23.0 | < 0.001* |  | 70.9 ± 24.5 | 56.8 ± 22.9 | < 0.001* |
| Number of  positive LN | 16.7 ± 20.9 | 18.3 ± 17.7 | 0.517 |  | 20.6 ± 22.4 | 17.5 ± 17.7 | 0.262 |
| Radicality |  |  | 0.854 |  |  |  | 0.822 |
| R0 | 141 (92.2%) | 119 (90.8%) |  |  | 94 (88.7%) | 96 (90.6%) |  |
| R1 | 12 (7.8%) | 12 (9.2%) |  |  | 12 (11.3%) | 10 (9.4%) |  |
| Proximal margin(cm) | 1.6 ± 1.9 | 1.7 ± 2.0 | 0.460 |  | 1.5 ± 1.9 | 1.7 ± 1.9 | 0.387 |
| Margin positive | 10 (6.5%) | 7 (5.3%) | 0.864 |  | 9 (8.5%) | 4 (3.8%) | 0.252 |
| Distal  margin(cm) | 4.9 ± 3.8 | 3.7 ± 3.8 | 0.014* |  | 4.0 ± 3.2 | 3.8 ± 3.3 | 0.738 |
| Margin positive | 4 (2.6%) | 7 (5.3%) | 0.379 |  | 3 (2.8%) | 6 (5.7%) | 0.498 |
| Splenectomy | 20 (13.1%) | 34 (26.0%) | 0.005* |  | 16 (15.1%) | 28 (26.4%) | 0.062 |
| pT |  |  | 0.020* |  |  |  | 0.526 |
| T1 | 2 (1.3%) | 0 (0.0%) |  |  | 0 (0.0%) | 0 (0.0%) |  |
| T2 | 8 (5.2%) | 0 (0.0%) |  |  | 2 (1.9%) | 0 (0.0%) |  |
| T3 | 32 (20.9%) | 23 (17.6%) |  |  | 17 (16.0%) | 19 (17.9%) |  |
| T4 | 111 (72.5%) | 108 (82.4%) |  |  | 87 (82.1%) | 87 (82.1%) |  |
| pN |  |  | 0.004* |  |  |  | 0.309 |
| N0 | 36 (23.5%) | 10 (7.6%) |  |  | 16 (15.1%) | 10 (9.4%) |  |
| N1 | 10 (6.5%) | 10 (7.6%) |  |  | 4 (3.8%) | 9 (8.5%) |  |
| N2 | 21 (13.7%) | 25 (19.1%) |  |  | 16 (15.1%) | 19 (17.9%) |  |
| N3 | 86 (56.2%) | 86 (65.6%) |  |  | 70 (66.0%) | 68 (64.2%) |  |
| pM |  |  | 0.671 |  |  |  | 0.814 |
| M0 | 140 (91.5%) | 117 (89.3%) |  |  | 95 (89.6%) | 97 (91.5%) |  |
| M1 | 13 (8.5%) | 14 (10.7%) |  |  | 11 (10.4%) | 9 (8.5%) |  |
| pStage |  |  | 0.006* |  |  |  | 0.888 |
| I | 5 (3.3%) | 0 (0.0%) |  |  | 1 (0.9%) | 0 (0.0%) |  |
| II | 35 (22.9%) | 14 (10.7%) |  |  | 14 (13.2%) | 14 (13.2%) |  |
| III | 100 (65.4%) | 103 (78.6%) |  |  | 80 (75.5%) | 83 (78.3%) |  |
| IV | 13 (8.5%) | 14 (10.7%) |  |  | 11 (10.4%) | 9 (8.5%) |  |
| Postoperative chemotherapy | 101 (66.0%) | 97 (74.0%) | 0.181 |  | 83 (78.3%) | 75 (70.8%) | 0.270 |
| Values are presented as mean ± standardized difference or n (%) unless otherwise indicated. | | | | | | | |
| *Statistically significant (P<0.05). | | | | | | | |
| ^†^Prior abdominal surgery included appendectomy, cholecystectomy, inguinal hernia repair, cesarean section and ovarian cystectomy. | | | | | | | |
| BMI indicates body mass index; ASA, American Society of Anesthesiologists, CCI, Charlson Comorbidity Index; LN, lymph node; pStage, pathologic stage. | | | | | | | |

| **Supplementary Table 4** Postoperative complication with OR and 95% CI in the LG and OG groups before and after PSM | | | | | | | |
| --- | --- | --- | --- | --- | --- | --- | --- |
|  | Before Propensity Score Matching | | |  | After propensity score matching | | |
|  | LG (n=153) | OG (n=131) | OR  (95% CI) |  | LG (n=106) | OG (n=106) | OR  (95% CI) |
| Early complication | 24 (15.7%) | 34 (26.0%) | 0.53  (0.30 – 0.95) |  | 22 (20.8%) | 31 (29.2%) | 0.63  (0.34 – 1.19) |
| Surgical complication |  |  |  |  |  |  |  |
| Wound | 0 (0.0%) | 6 (4.6%) | - |  | 0 (0.0%) | 6 (5.7%) | - |
| Bleeding | 2 (1.3%) | 3 (2.3%) | 1.72  (0.15 – 19.21) |  | 2 (1.9%) | 1 (0.9%) | 2.02  (0.18 – 22.61) |
| Intra-abdominal Infection | 5 (3.3%) | 16 (12.2%) | 0.24  (0.09 – 0.68) |  | 3 (2.8%) | 15 (14.2%) | 0.18  (0.05 – 0.63) |
| Anastomosis leakage | 2 (1.3%) | 3 (2.3%) | 0.57  (0.09 – 3.44) |  | 1 (0.9%) | 2 (1.9%) | 0.50  (0.04 – 5.55) |
| Stump leakage | 0 (0.0%) | 1 (0.8%) | - |  | 0 (0.0%) | 1 (0.9%) | - |
| Postoperative  pancreatic fistula | 1 (0.7%) | 4 (3.1%) | 0.21  (0.02 – 1.89) |  | 1 (0.9%) | 4 (3.8%) | 0.24  (0.03 – 2.21) |
| Intra-abdominal abscess | 2 (1.3%) | 8 (6.1%) | 0.20  (0.04 – 0.98) |  | 1 (0.9%) | 8 (7.5%) | 0.12  (0.01 – 0.95) |
| Anastomosis stenosis | 2 (1.3%) | 0 (0.0%) | - |  | 2 (1.9%) | 0 (0.0%) | - |
| Motility disorder | 3 (2.0%) | 3 (2.3%) | 0.85  (0.17 – 4.30) |  | 3 (2.8%) | 2 (1.9%) | 1.51  (0.25 – 9.25) |
| Medical complication |  |  |  |  |  |  |  |
| Pulmonary | 7 (4.6%) | 2 (1.5%) | 3.09  (0.63 – 15.15) |  | 7 (6.6%) | 2 (1.9%) | 3.68  (0.75 – 18.13) |
| Urinary | 1 (0.7%) | 2 (1.5%) | 0.42  (0.04 – 4.73) |  | 1 (0.9%) | 2 (1.9%) | 0.50  (0.04 – 5.55) |
| Gastrointestinal | 1 (0.7%) | 0 (0.0%) | - |  | 1 (0.9%) | 0 (0.0%) |  |
| Hepatobiliary | 2 (1.3%) | 1 (0.8%) | 1.72  (0.15 – 19.21) |  | 2 (1.9%) | 1 (0.9%) | 2.02  (0.18 – 22.61) |
| Cardiac | 1 (0.7%) | 0 (0.0%) | - |  | 1 (0.9%) | 0 (0.0%) | - |
| Neuropsychiatric | 0 (0.0%) | 1 (0.8%) | - |  | 0 (0.0%) | 1 (0.9%) | - |
| Vascular | 0 (0.0%) | 2 (1.5%) | - |  | 0 (0.0%) | 1 (0.9%) | - |
| Severity |  |  |  |  |  |  |  |
| Overall complication,  C-D ≥ II | 23 (15.0%) | 28 (21.4%) | 0.65  (0.35 – 1.20) |  | 21 (19.8%) | 25 (23.6%) | 0.80  (0.42 – 1.54) |
| Surgical complication | 11 (7.2%) | 21 (16.0%) | 0.41  (0.19 – 0.88) |  | 9 (8.5%) | 19 (17.9%) | 0.43  (0.18 – 0.99) |
| Medical complication | 12 (7.8%) | 7 (5.3%) | 1.51  (0.58 – 3.95) |  | 12 (11.3%) | 6 (5.7%) | 2.13  (0.77 – 5.90) |
| Overall complication,  C-D ≥ IIIa | 13 (8.5%) | 15 (11.5%) | 0.72  (0.33 – 1.57) |  | 12 (11.3%) | 16 (15.1%) | 0.72  (0.32 – 1.60) |
| Surgical complication | 7 (4.6%) | 14 (10.7%) | 0.40  (0.16 – 1.03) |  | 6 (5.7%) | 15 (14.2%) | 0.36  (0.14 – 0.98) |
| Medical complication | 6 (3.9%) | 1 (0.8%) | 5.31  (0.63 – 44.66) |  | 6 (5.7%) | 1 (0.9%) | 6,30  (0.45 – 53.26) |
| Late complication | 7 (4.6%) | 5 (3.8%) | 1.21  (0.37 – 3.90) |  | 5 (4.7%) | 4 (3.8%) | 0.73  (0.33 – 4.84) |
| Anastomosis stricture | 2 (1.3%) | 1 (0.8%) | 1.72  (0.15 – 19.21) |  | 1 (0.9%) | 1 (0.9%) | 1.00  (0.06 – 16.20) |
| Anastomosis leakage | 0 (0.0%) | 1 (0.8%) | - |  | 0 (0.0%) | 1 (0.9%) | - |
| Adhesive ileus | 2 (1.3%) | 2 (1.5%) | 0.854  (0.12 – 6.15) |  | 1 (0.9%) | 1 (0.9%) | 1.00  (0.06 – 16.20) |
| Internal hernia | 1 (0.7%) | 0 (0.0%) | - |  | 1 (0.9%) | 0 (0.0%) | - |
| Incisional hernia | 1 (0.7%) | 0 (0.0%) | - |  | 1 (0.9%) | 0 (0.0%) | - |
| Aorto-esophagojejunostomy  fistula | 0 (0.0%) | 1 (0.8%) | - |  | 0 (0.0%) | 1 (0.9%) | - |
| Entero-cutaneous fistula | 1 (0.7%) | 0 (0.0%) | - |  | 1 (0.9%) | 0 (0.0%) | - |
| Severity |  |  |  |  |  |  |  |
| C-D ≥ II | 7 (4.6%) | 5 (3.8%) | 1.21  (0.37 – 3.90) |  | 5 (4.7%) | 4 (3.8%) | 1.26  (0.33 – 4.84) |
| C-D ≥ IIIa | 5 (3.3%) | 4 (3.1%) | 1.07  (0.28 – 4.08) |  | 3 (2.8%) | 3 (2.8%) | 1.00  (0.20 – 5.07) |
| Values are presented as n (%). | | | | | | | |
| *Statistically significant (P<0.05). | | | | | | | |
| LG indicates laparoscopic gastrectomy; OG, open gastrectomy; OR, odds ratio; CI, confidence interval; PSM, propensity score matching; C-D, Clavien-Dindo classification. | | | | | | | |

| **Supplementary Table 5** The first recurrence pattern with OR and 95% CI of the LG and OG groups (after PSM) | | | |
| --- | --- | --- | --- |
|  | LG (N=95) | OG (N=97) | OR (95% CI) |
| Recurrence | 62 (65.3%) | 69 (71.1%) | 0.76  (0.42-1.40) |
| Anastomosis site | 1 (1.1%) | 2 (2.1%) | 0.51  (0.05-5.67) |
| Peritoneum | 46 (48.4%) | 67 (69.1%) | 0.42  (0.23-0.76) |
| Retroperitoneum | 16 (16.8%) | 17 (17.5%) | 0.95  (0.45-2.02) |
| Distant lymph node^†^ | 11 (11.6%) | 9 (9.3%) | 1.28  (0.51-3.25) |
| Liver | 1 (1.1%) | 1 (1.0%) | 1.02  (0.06-16.57) |
| Lung | 2 (2.1%) | 2 (2.1%) | 1.02  (0.14-7.40) |
| Pleura | 2 (2.1%) | 2 (2.1%) | 1.02  (0.14-7.40) |
| Bone | 3 (3.2%) | 2 (2.1%) | 1.55  (0.25-9.48) |
| Ovary | 2 (2.1%) | 3 (3.1%) | 0.67  (0.11-4.13) |
| Multiple recurrence sites^‡^ | 44 (46.3%) | 47 (48.5%) | 0.92  (0.52-1.62) |
| Values are presented as n (%). | | | |
| ^†^Distant lymph nodes are defined as lymph nodes located outside D2 area according to the Japanese Gastric Cancer Association guideline^19^. | | | |
| ^‡^Multiple recurrence is defined as the presence of more than one site of recurrence at the time of initial detection. | | | |
| LG indicates laparoscopic gastrectomy; OG, open gastrectomy; PSM, propensity score matching; OR, odds ratio; CI, confidence interval. | | | |

| **Supplementary Table 6** Subgroup analysis for adjuvant chemotherapy by era | | | | | | | | | | | |
| --- | --- | --- | --- | --- | --- | --- | --- | --- | --- | --- | --- |
|  | 2003 -2007 | | |  | 2008 - 2012 | | |  | 2013 - 2019 | | |
|  | LG  (n=1) | OG  (n=46) | *P* |  | LG  (n=28) | OG  (n=33) | *P* |  | LG  (n=66) | OG  (n=18) | *P* |
| Adjuvant chemotherapy (%) | 1 (100%) | 29 (63.0%) | 1.000 |  | 20 (71.4%) | 22 (66.7%) | 0.902 |  | 53 (80.3%) | 15 (83.3%) | 1.000 |
| 5-year OS rate (%) | 0.0% | 33.3% | 0.007* |  | 25.5% | 30.0% | 0.694 |  | 42.8% | 0.0% | 0.001* |
| 5-year RFS rate (%) | 0.0% | 26.1% | 0.035* |  | 17.9% | 21.2% | 0.479 |  | 16.7% | 0.0% | <0.001* |
| Peritoneal recurrence rate (%) | 100% | 58.7% | 0.019* |  | 57.1% | 51.5% | 0.553 |  | 43.9% | 94.4% | <0.001* |
| Values are presented as n (%) unless otherwise indicated. | | | | | | | | | | | |
| *Statistically significant (*P*<0.05) | | | | | | | | | | | |
| LG indicates laparoscopic gastrectomy; OG, open gastrectomy; OS, overall survival; RFS, recurrence-free survival. | | | | | | | | | | | |

**
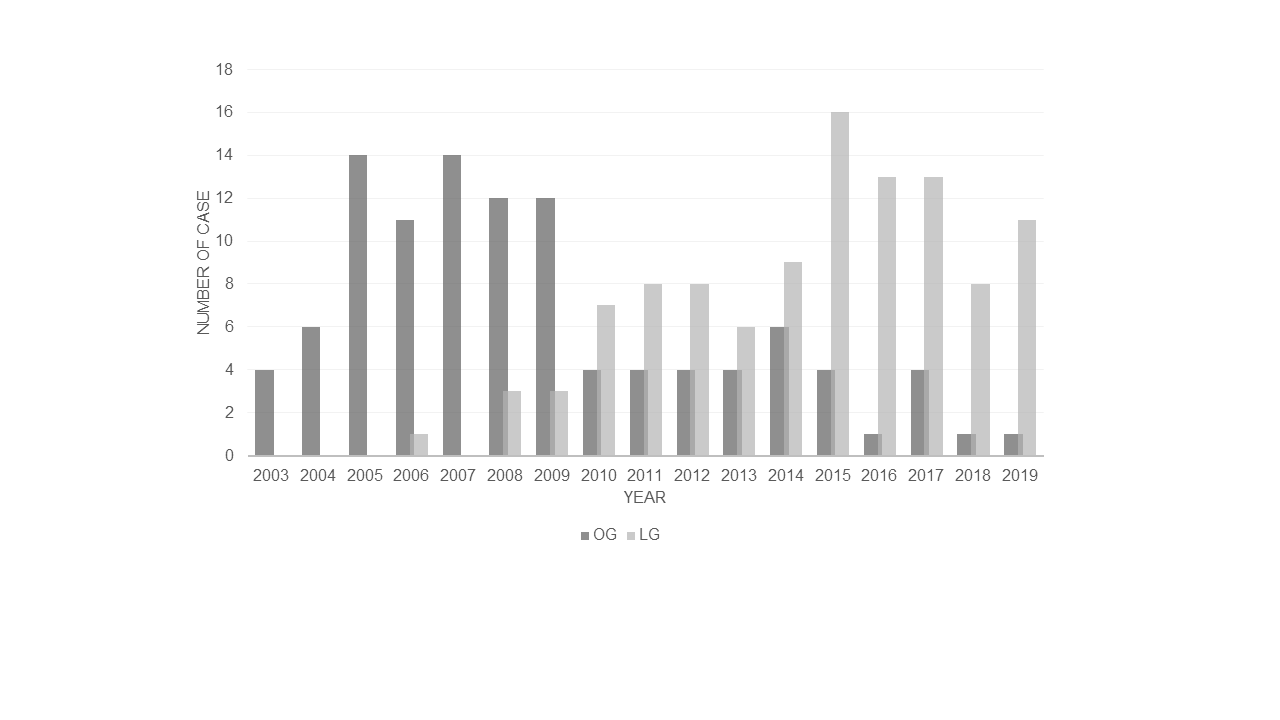
Supplementary Figure 1** Change in the number of case of the LG and OG by year

**Supplementary Figure 2** Kaplan-Meier curves of 5-year OS by era in LG and OG groups


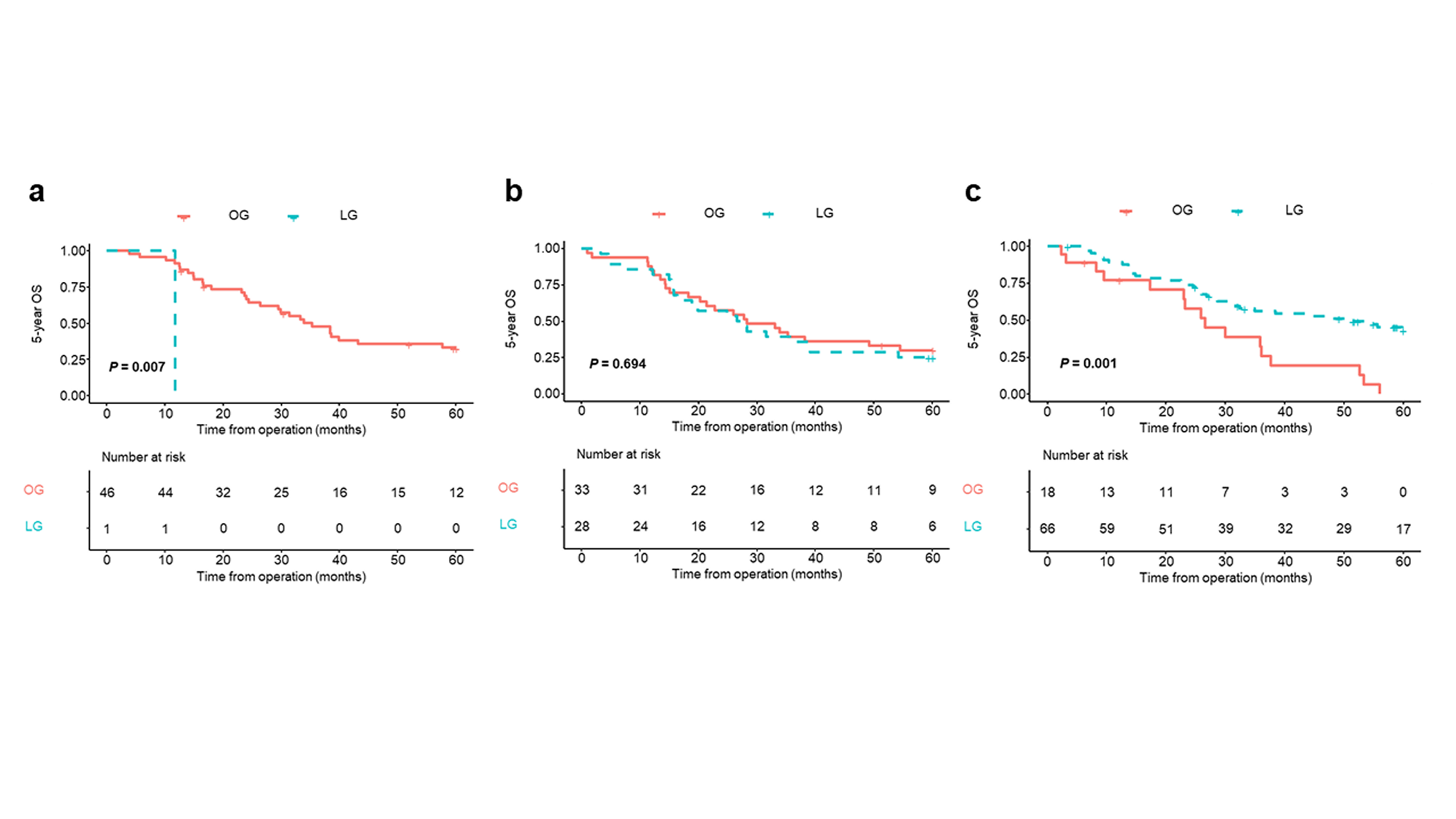


Kaplan–Meier survival curves comparing 5-year overall survival (OS) between laparoscopic gastrectomy (LG) and open gastrectomy (OG) across three clinical eras: **(a)** 2003–2007, **(b)** 2008–2012, and **(c)** 2013–2019

**Supplementary Figure 3** Kaplan-Meier curves of 5-year RFS by era in LG and OG groups


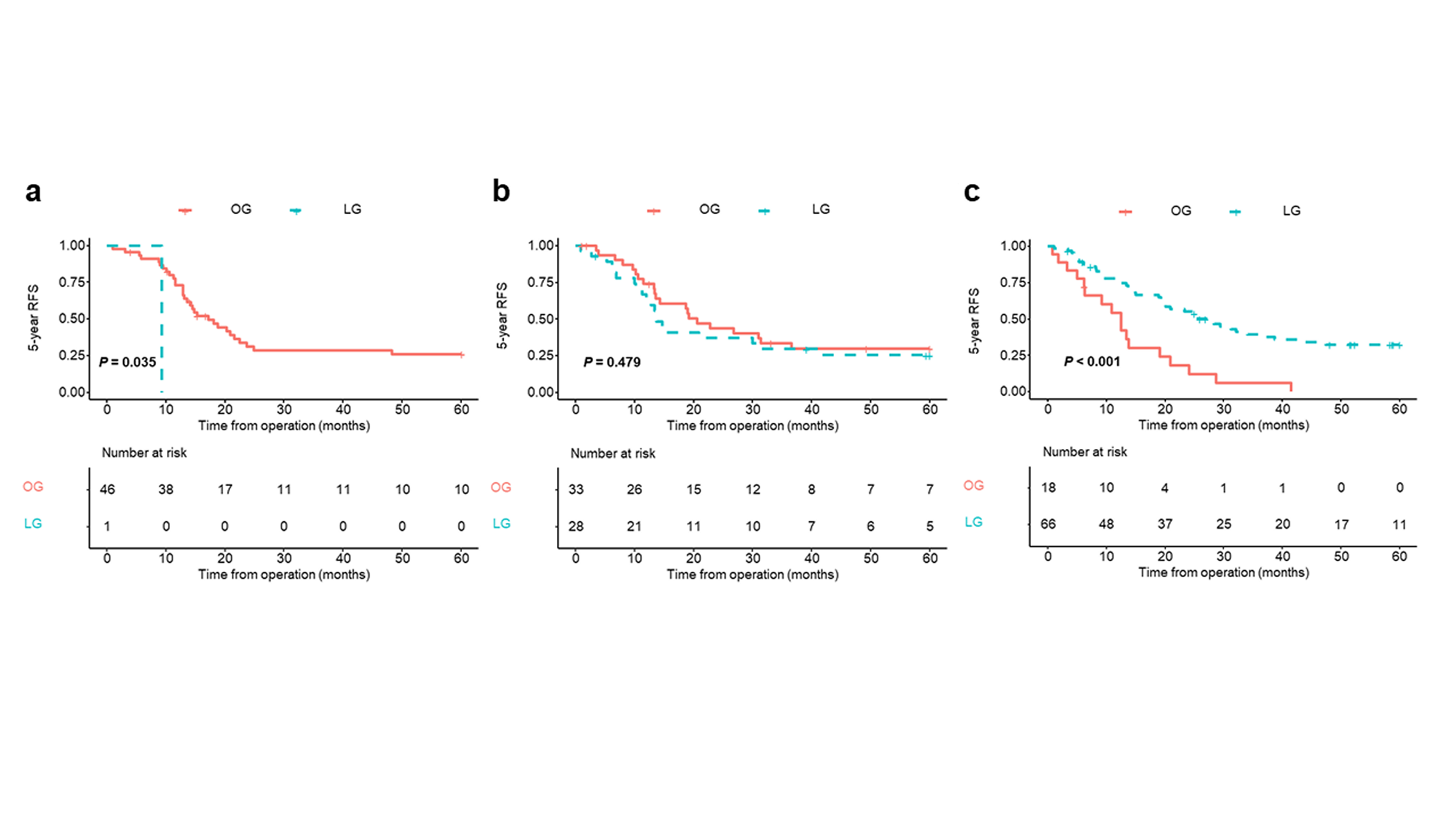


Kaplan–Meier survival curves comparing 5-year recurrence-free survival (RFS) between laparoscopic gastrectomy (LG) and open gastrectomy (OG) across three clinical eras: **(a)** 2003–2007, **(b)** 2008–2012, and **(c)** 2013–2019

**Supplementary Figure 4** Cumulative incidence of peritoneal recurrence by era in LG and OG groups


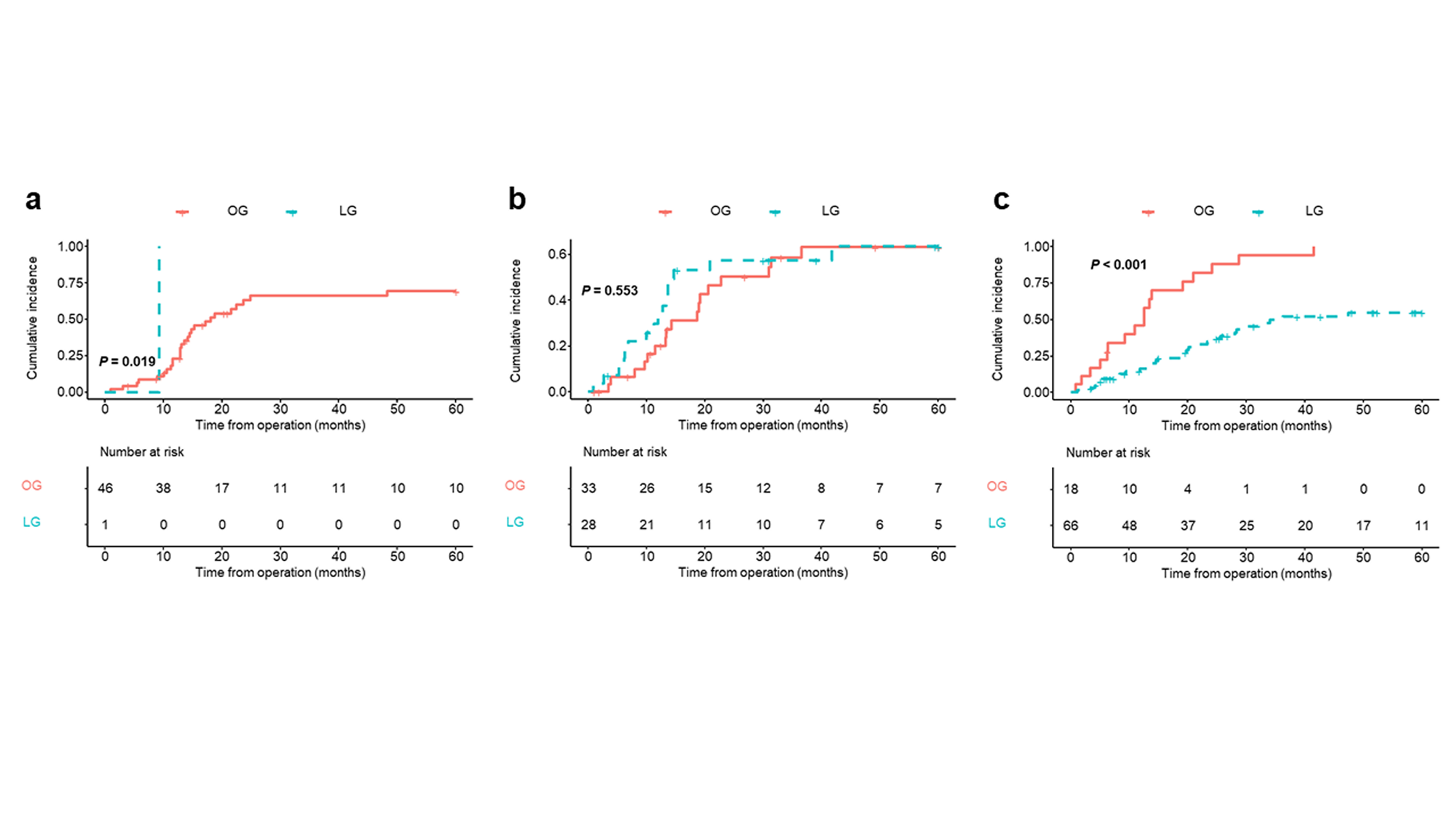


Cumulative incidence curves comparing peritoneal recurrence rates between laparoscopic gastrectomy (LG) and open gastrectomy (OG) across three time periods: **(a)** 2003–2007, **(b)** 2008–2012, and **(c)** 2013–2019
